# Supplementary material for: Resolving the full spectrum of human genome variation using Linked-Reads
Source: Genome Res. 2019 Apr;29(4):635–45. doi: 10.1101/gr.234443.118 (PMC6442396; doi:10.1101/gr.234443.118)
Supplement: Supplemental Material [file supp_gr.234443.118_Supplemental_longranger-2.2.zip › longranger-2.2.tar.bz2!/lr2.2-public/lib/stan/cmdstan/stan/doc/api/html/arg__sample__algo_8hpp_source.html]

Stan: src/stan/gm/arguments/arg\_sample\_algo.hpp Source File


|  |  |
| --- | --- |
|  | Stan  2.5.0  probability, sampling & optimization |


- Main Page
- Related Pages
- Namespaces
- Classes
- Files

- File List
- File Members

All Classes Namespaces Files Functions Variables Typedefs Enumerations Enumerator Friends Macros Pages

- src
- stan
- gm
- arguments

arg\_sample\_algo.hpp

Go to the documentation of this file.

1 #ifndef STAN\_\_GM\_\_ARGUMENTS\_\_SAMPLE\_\_ALGO\_\_HPP

2 #define STAN\_\_GM\_\_ARGUMENTS\_\_SAMPLE\_\_ALGO\_\_HPP

3

4 #include <stan/gm/arguments/list\_argument.hpp>

5

6 #include <stan/gm/arguments/arg\_hmc.hpp>

7 #include <stan/gm/arguments/arg\_fixed\_param.hpp>

8

9 namespace stan {

10

11  namespace gm {

12

13  class arg\_sample\_algo: public list\_argument {

14

15  public:

16

17  arg\_sample\_algo() {

18

19  \_name = "algorithm";

20  \_description = "Sampling algorithm";

21

22  \_values.push\_back(new arg\_hmc());

23  \_values.push\_back(new arg\_fixed\_param());

24

25  \_default\_cursor = 0;

26  \_cursor = \_default\_cursor;

27

28  }

29

30  };

31

32  } // gm

33

34 } // stan

35

36 #endif

37

stan::gm::arg\_hmc

**Definition:** arg\_hmc.hpp:15

arg\_fixed\_param.hpp

stan::gm::list\_argument::\_default\_cursor

int \_default\_cursor

**Definition:** list\_argument.hpp:183

list\_argument.hpp

stan::gm::list\_argument::\_values

std::vector< argument \* > \_values

**Definition:** list\_argument.hpp:185

stan::gm::arg\_fixed\_param

**Definition:** arg\_fixed\_param.hpp:10

stan::gm::list\_argument

**Definition:** list\_argument.hpp:12

stan::gm::argument::\_name

std::string \_name

**Definition:** argument.hpp:76

stan::gm::list\_argument::\_cursor

int \_cursor

**Definition:** list\_argument.hpp:182

arg\_hmc.hpp

stan::gm::arg\_sample\_algo

**Definition:** arg\_sample\_algo.hpp:13

stan::gm::arg\_sample\_algo::arg\_sample\_algo

arg\_sample\_algo()

**Definition:** arg\_sample\_algo.hpp:17

stan::gm::argument::\_description

std::string \_description

**Definition:** argument.hpp:77


---

[ Stan Home Page ]

*© 2011–2014,
Stan Development Team.*
